# Supplementary material for: Soybean reduced internode 1 determines internode length and improves grain yield at dense planting
Source: Nat Commun. 2023 Dec 1;14:7939. doi: 10.1038/s41467-023-42991-z (PMC10692089; doi:10.1038/s41467-023-42991-z)
Supplement: Supplementary file 1 — Supplementary Information [file 41467_2023_42991_MOESM1_ESM.pdf]

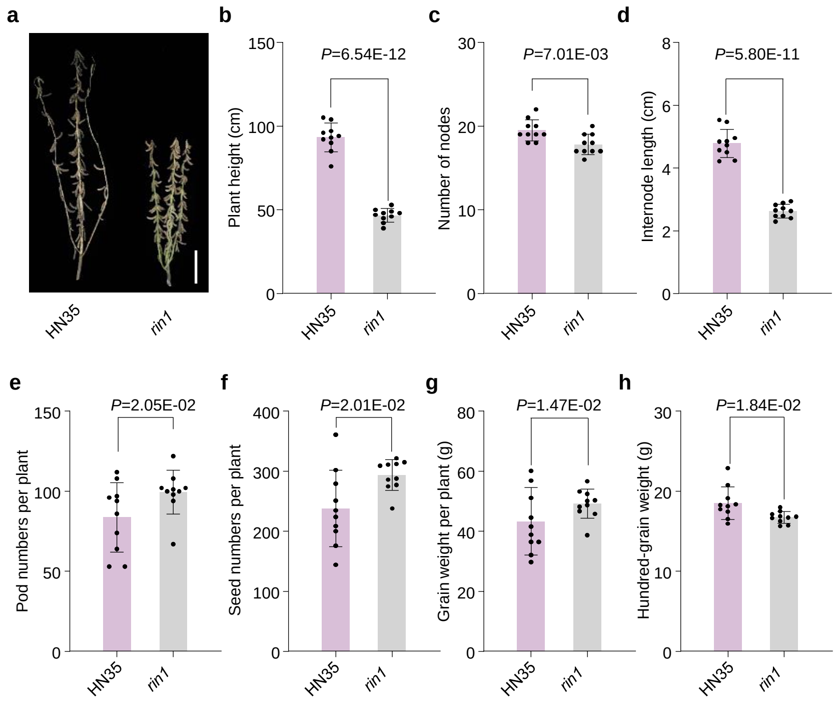

Supplementary Fig. 1 Phenotypic differences between wild-type HN35 and its mutant *rin1* in the field in Harbin, 2017.
a Phenotype of HN35 and *rin1* at maturity. Scale bar = 10 cm. **b** Plant height (cm). **c** Number of nodes. **d** Internode length (cm). **e** Pod numbers per plant. **f** Seed numbers per plant. **g** Grain weight per plant (g). **h** Hundred-grain-weight per plant (g). All plants were planted in a nature field of Harbin, China (45°75′N, 126°63′E) and the phenotypes were scored after maturity. All data of (**b**)–(**h**) are means ± SEM (n = 10 plants). A student's *t*-test (two-sided) was used to generate the *P* values.

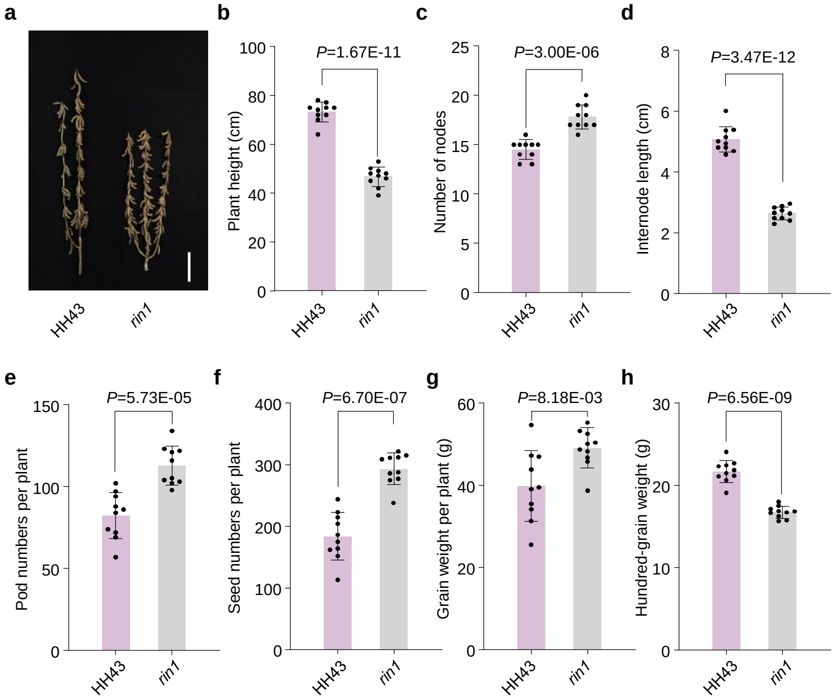

Supplementary Fig. 2 Phenotypic differences between HH43 and *rin1* in the field in Harbin, 2017.
a Phenotype of HH43 and *rin1* at maturity. Scale bar = 10 cm. **b** Plant height (cm). **c** Number of nodes. **d** Internode length (cm). **e** Pod numbers per plant. **f** Seed numbers per plant. **g** Grain weight per plant (g). **h** Hundred-grain-weight per plant (g). All plants were planted in a nature field of Harbin, China (45°75′N, 126°63′E) and the phenotypes were scored after maturity. All data of (**b**)–(**h**) are means ± SEM (n = 10 plants). A student's *t*-test (two-sided) was used to generate the *P* values.

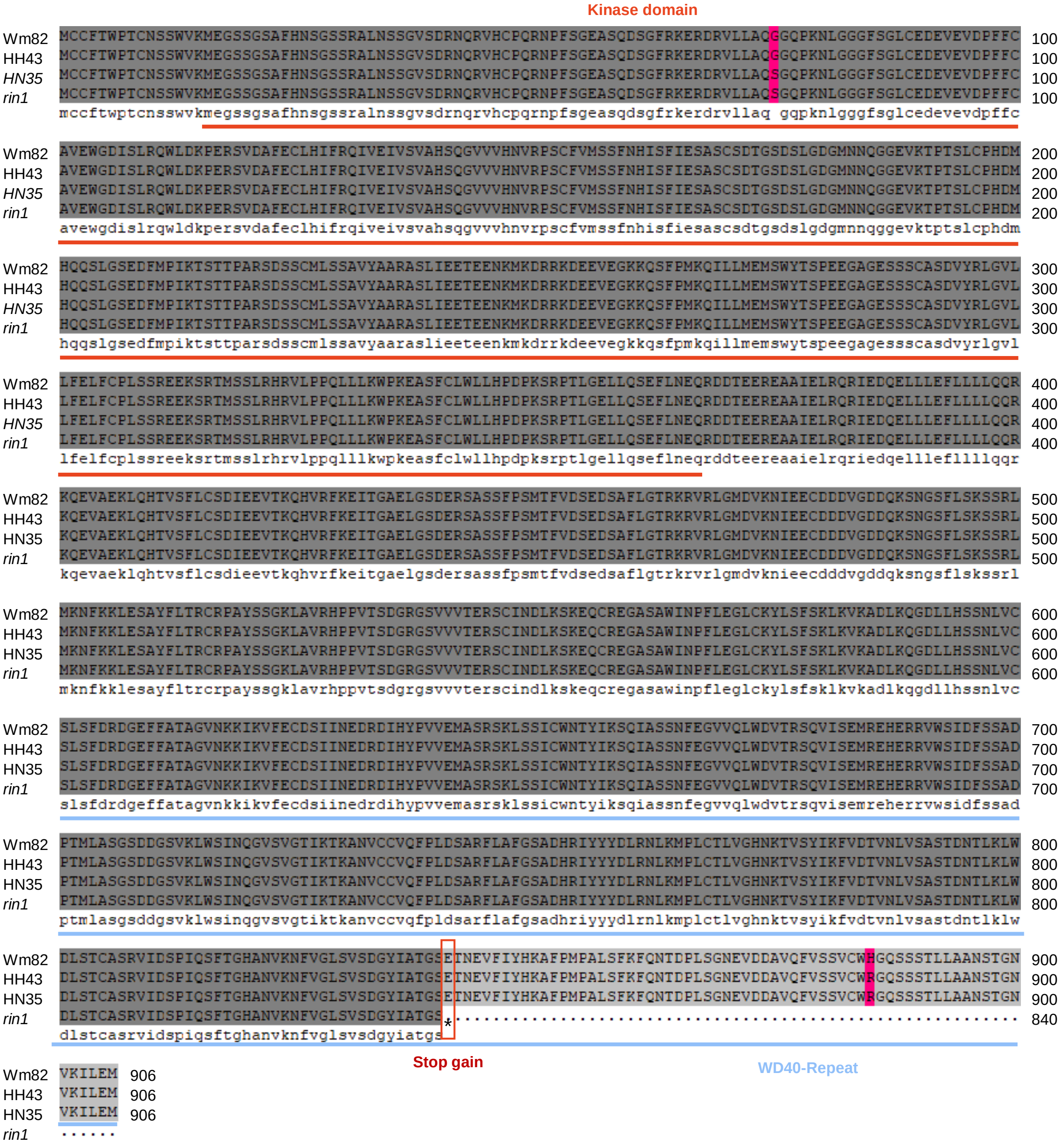

Supplementary Fig. 3 Alignment of soybean RIN1 sequences between the reference accession Wm82, HH43, HN35, and *rin1*.

Amino-acid sequences were aligned by ClustalW Multiple alignment in DNAMAN. Three amino-acid differences were detected in the kinase domain and WD40-repeat domain, respectively. The red line represents kinase domain and blue line represents WD40-repeat domain. The pink box represents the amino acid variant position. The asterisk indicates stop-codon gain.

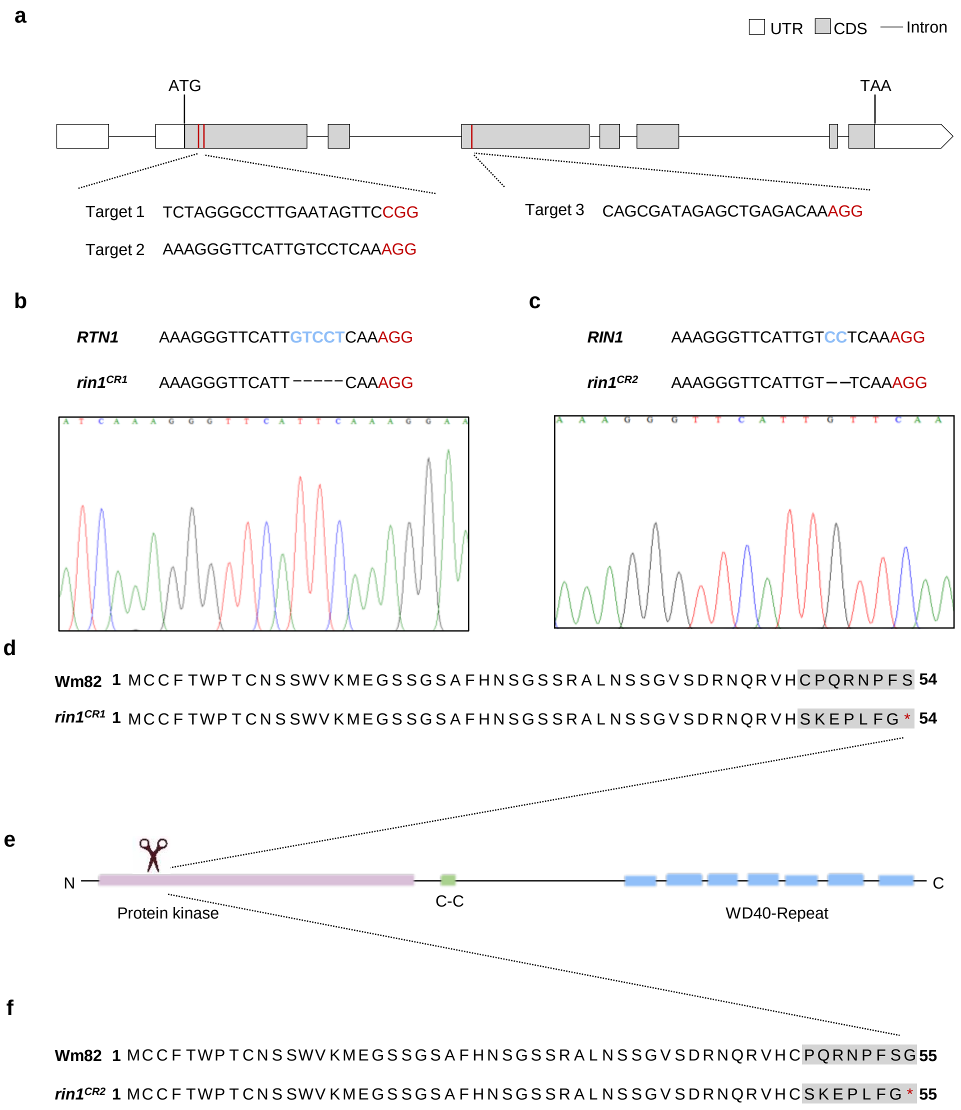

Supplementary Fig. 4 CRISPR/Cas9 targets and proteins of RIN1 in *rin1*^{CR} mutants.
a The position of three editing targets in *RIN1*. The red lines indicate the CRISPR/Cas9 targets. **b**,**c** Sanger sequencing reveals a 5-bp deletion at target site 2, designated as *rin1*^{CR1} (**b**) and a 2-bp deletion at target site 2 is designated as *rin1*^{CR2} (**c**). The picture of (**b**) and (**c**) were generated by BioEdit. **d** Protein sequence of RIN1 in Wm82 and *rin1*^{CR1}. **e** Schematic of functional domains of RIN1. The scissor represents the protein termination position. **f** Protein sequence of RIN1 in Wm82 and *rin1*^{CR2}. The gray highlighting in (**d**) and (**f**) depicts the amino acids in *rin1*^{CR} mutants that differ from wild-type Wm82. The asterisk indicates stop codon.

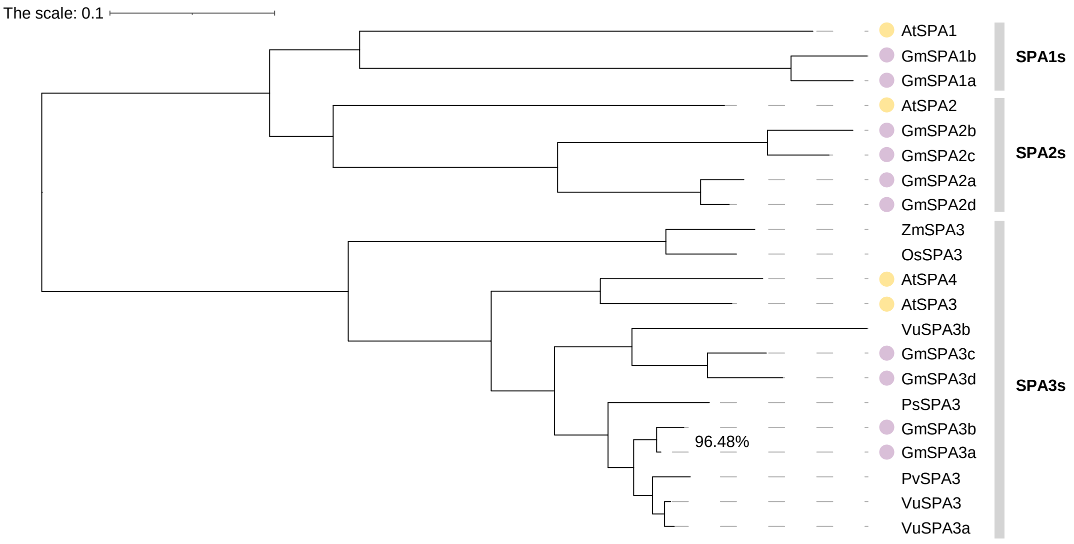

Supplementary Fig. 5 Phylogenetic tree of the SPA proteins from Arabidopsis, soybean, rice, maize and certain leguminous crops.

There are 10 *SPA* genes in soybean, including *SPA1a* (*Glyma.07G058200*), *GmSPA1b* (*Glyma.16G027200*), *GmSPA2a* (*Glyma.08G021200*), *GmSPA2b* (*Glyma.05G214900*), *GmSPA2c* (*Glyma.11G018700*), *GmSPA2d* (*Glyma.01G224200*), *GmSPA3a* (*Glyma.12G224600*), *GmSPA3b* (*Glyma.13G276700*), *GmSPA3c* (*Glyma.06G242100*) and *GmSPA3d* (*Glyma.12G155700*), which cluster into three clades that we designate SPA1s, SPA2s and SPA3s. Phylogenetic tree of SPA proteins from *Arabidopsis* (*At*), *Glycine max* (*Gm*), *Pisum sativum* (*Ps*), *Phaseolus vulgaris* (*Pv*), *Vigna unguiculata* (*Vu*), *Oryza sativa* (*Os*), *Zea mays* (*Zm*) using the Neighbor-Joining Method by MEGA6 software. The protein homology between GmSPA3a and GmSPA3b is 96.48%. The purple circles represent the SPA in soybeans and the yellow circles represent the SPA in Arabidopsis. All amino-acid sequences are listed in Source Data file.

```
                                                                                Target 1                          Target 2
Glyma.12G224600   GGTGAAGATGGAGGGTTCTTCTGGGTCTGCTTTTCACAATTCTGGCAGTTCTAGGGCCTTGAATAGTTCCGGAGTCTCAGATAGGAATCAAAGGGTTCAT   138
Glyma.13G276700   GATGAAGATGGAG...CCTTCTGGGTCTGCTTTTCAGAATTCTGGCAGTTCTAGGGCCTTGAACAGTTCTGGAGTCTCAGATAGGAACCAAAGGGTTCAT   135
Glyma.06G242100   ....................................................................................................     0
Glyma.12G155700   ....................................................................................................     0
Glyma.16G027200   AATGCACATCTAATTAGCAGTATAACTCAGAGTA.CTTCATCTGCATATAATTATCCTCAATTGATTGTAAAGCAAACAAAGAAGGGAAAGGGGATTATT   609
Glyma.07G058200   AATGCACATCTTATTAGCAGTATAACTCAGAGTA.CTTCATCTGCATATAATTATCCTCAATTGATTGTAAAGCAAACAAGGAAGGGAAAAGGGGTTATT   642
Glyma.01G224200   GTCATTGATGCGGATCAGAATCAGCTAAAGACTGGCATTG.ATGCGGATCAGAATCGGCTAAAGACTGGCATTGATGCAGACCAGAATCAGATGAAGGCT   894
Glyma.05G214900   ..............................................................................................ATGACG     6
Glyma.11G018700   GTCATTGATGTGATTCAGAATCGGCTGAAGACTGGCATTG.ATGCAGATCAGAATCCGATGAAGACTGGCATTGAT......CAGAGTCGGATGAAGACC   876
Glyma.08G021200   GATGAAGGTCATGCTCACGAAGGAATACAAACAAAAGTTATACACAAATCAGGATTTGCAGAGTATTCTGGTAGAAGTACATTGAAGGGAAAGGGTGTTG   622
Consensus
                    Target 2
Glyma.12G224600   TGTCCTCAAAGGAACCCCTTTTCGGGTGAGGCATCACAGGATTCGGGGTTTAGAAAGGAAAGGGATAGGGTTCTGTTGGCTCAAGGTGGTCAGCCTAAAA   238
Glyma.13G276700   TGTCCTCAAAGGAACCCCTTCTTGGGTGAGGCATCCCAGGATTCGGGGTTCAGAAAGGAAAGGGATAGGTTTCTGTTGGCTCAAGGTGGTCAGCCTAAGA   235
Glyma.06G242100   ....................................................................................................     0
Glyma.12G155700   ....................................................................................................     0
Glyma.16G027200   TGTGAAGATTTAAACCAAAGCTTCAGTACTGGAGGAGCACTTAACAG..CCAAGAGGATGAGAAACCTGCTTTTGCAGCTAAGTTTCAATCCGAGACACT   707
Glyma.07G058200   TGTGAAGATTTAAACAAAAGCTTCAGTATTGGAGGAGCACTTAAGAG..TCAAGAGGATGAGAAACTTGGTTTTGCCGCTAAGTTTCAATCCGAGACACT   740
Glyma.01G224200   AGCATTGGCACAGATCAGAATCAGATGAAGAATCACAGTGGTACTGA..TCAGAAACAGATGAAGACTGGCATTGTTACTCACTTGAACTCTAATCAATC   992
Glyma.05G214900   TCTCTAACAAGTTATGAAGTAGTAGTTTTATTATCCGGTG.TGCGG....TGGAACCTGATAAAGTCTGGCATTGACACTCAGATGGATTCTAAGGCATT   101
Glyma.11G018700   GGCATTGATACAGATCAGAATCAGATGAAAACTGGCATTGGTACTGA..TCAGAAACAGATGAAGACCAGCATTGGTACTCACTTGAACTCTAATCAATC   974
Glyma.08G021200   TATGTAAAGGTCCATCTTCTAATGGCTTATATATTGAGTC.TAGAGA..TCAGAACCCGATAAAGTCTGGTATTGACACTCAGATGGATTCTAATGCATT   719
                                                 Target 3
Glyma.12G224600   AACAGAGAGATGATACGGAAGAACGTGAAGCAGCGATAGAGCTGAGACAAAGGATA.GAGGATCAGGAGTTGTTGTTAGAGTTCCTTTTGTTACTTCAAC  1195
Glyma.13G276700   AACAGAGAGATGATATGGAAGAACGTGAAGCAGCGATAGAGCTGAGACAAAGGATA.GATGATCAGGAGTTGCTGTTAGAATTCCTTTTATTACTTCAAC  1195
Glyma.06G242100   AACCAAGAGATGATATAGAAAAGTGTGAAGCAGTGGTAGAAATTGGAGAAAGGATA.GATGACCAGGAGTTGTTGCTAGAATTCCTTTCGTTAATTCAAC   874
Glyma.12G155700   AACCAAGAGATGATATAGAAAACTGTGAGGCAGCGGTAGAAGTTAGAGAAAGGATA.GATGATCAGGAGTTGTTGCAAGAATTCCTTTCATTAATTCAAC   871
Glyma.16G027200   AATCAGAGGA....ATCAAATTCTGTAGA.TGATGTTGGAATATC..TGATGACGAGGCTGAAACAGAGCAATTACTAGATTTTCTAATTTTAATCAAAG  1630
Glyma.07G058200   AATCAAAGGA....ATCAAATTCTGTAGA.TGATGTTGGAATATC..TGATGATGAGGCTGAAACAGAGCAATTACTAGATTTTCTAATTTTATTCAAAG  1666
Glyma.01G224200   GATTGCAAGA....GTTGTTTAGTGAGGAATTGTCATCAAGTATTGACCAAGAAGATGCAGAATCTGAATTGTTATTGCATTTCCTCGTTTTGTTAAAAG  1963
Glyma.05G214900   GAATGCGGGA....GGTGTACTTTGAGGAATTATCATTAAGTCTTAATCAAGATGATGCAGAATCAGAATTGCTATTGCATTTCCTCATCTCACTAGAAG  1090
Glyma.11G018700   GATTGCAAGA....GTTGTTTAGTGAGGAATTGTCATCAAGTATTGACCAAGAAGATGCAGAATCAGAATTGTTATTGCATTTCCTCGTTTTGTTAAAAG  1945
Glyma.08G021200   GAATCCATGA....GGTGTACTGTGAGGAATTATCATCAAGTCTCAATCAAGATGATGCAGAATCAGAATTGCTATTGCATTTCCTCATCTCACTAAAAG  1708
```

Supplementary Fig. 6 The similarity comparison between 10 *RIN1* homologous genes in soybean for *RIN1* CRISPR/Cas9 targets.

The blue line, red line and green line represents the Target 1, Target 2 and Target 3, respectively. *SPA3b* (*Glyma.13G276700*) gene shares the same sequences with the Target 2 and Target 3 of *rin1*^{CR}. In the Target 1, SPA3b differs from *rin1*^{CR} with a SNP. Nucleic acid sequences were aligned by DNAMAN.

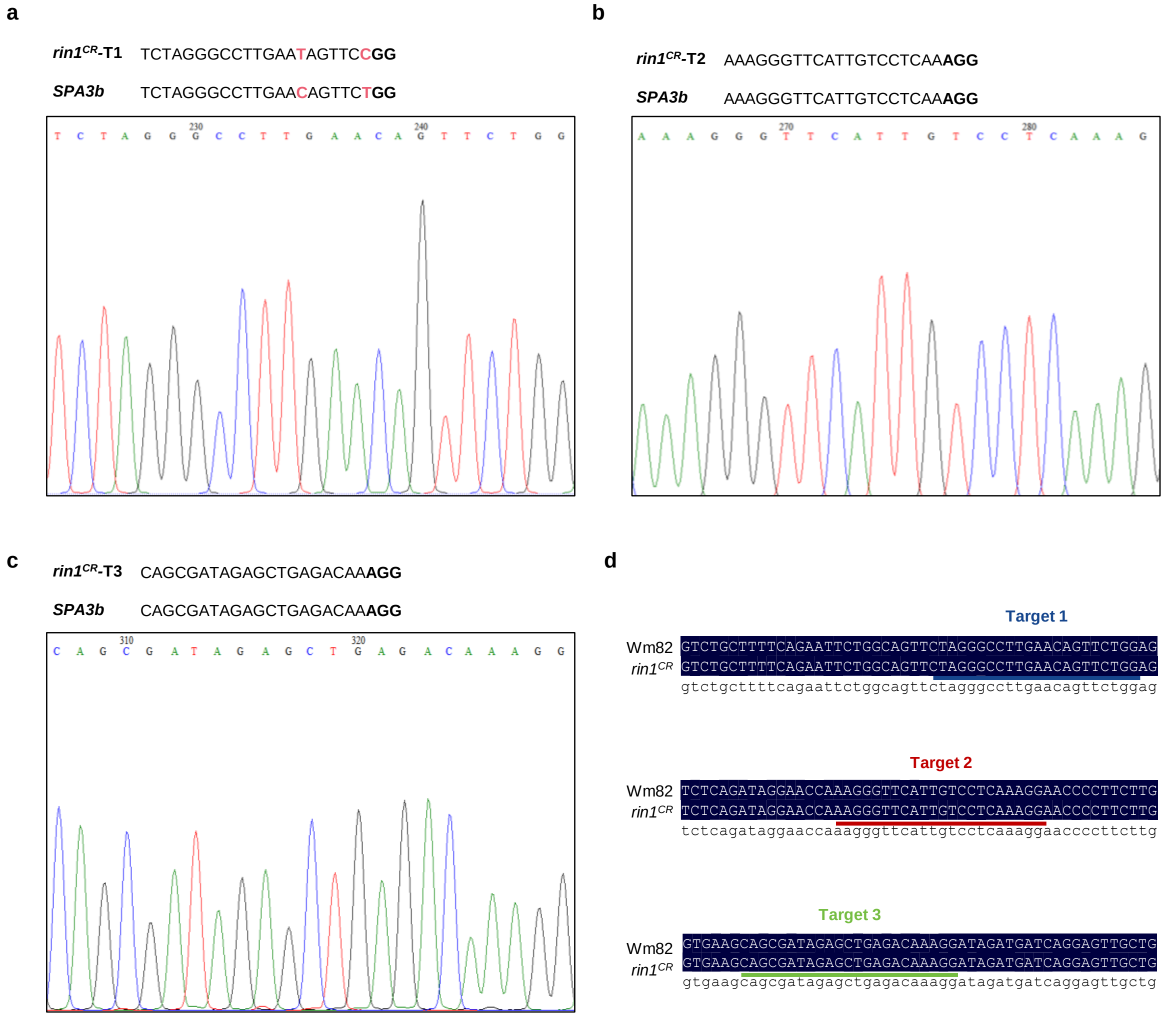

Supplementary Fig. 7 The nucleic acid sequences of *SPA3b* gene in the position of *rin1^{CR}* targets in Wm82 and *rin1^{CR}*.

a The nucleic acid sequence of *SPA3b* gene in the position of *rin1^{CR}* Target 1 (*rin1^{CR}*-T1). **b** The nucleic acid sequence of *SPA3b* gene in the position of *rin1^{CR}* Target 2 (*rin1^{CR}*-T2). **c** The nucleic acid sequence of *SPA3b* gene in the position of *rin1^{CR}* Target 3 (*rin1^{CR}*-T3). The picture of (**a**), (**b**) and (**c**) were generated by BioEdit. **d** The nucleic acid sequences of *SPA3b* gene in the position of *rin1^{CR}* targets in Wm82 and *rin1^{CR}*. The nucleic acid sequences were aligned by DNAMAN. The blue line, red line and green line represents the Target 1, Target 2 and Target 3, respectively.

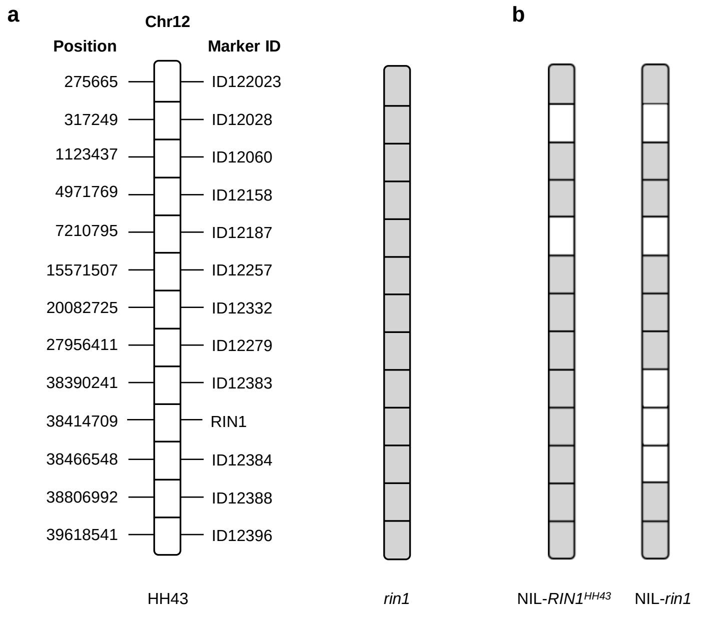

Supplementary Fig. 8 The construction of *RIN1* NILs derived from the cross between HH43 and *rin1*.
a Molecular markers and their genomic positions in HH43 (left) and *rin1* (right) on chromosome 12. **b** Introgression segments of *RIN1* in NIL-*RIN1*^{HH43} (left) and NIL-*rin1* (right). White represents the segments derived from HH43, and gray represents the segments derived from *rin1*. Marker sequences are listed in Supplementary Table 4.

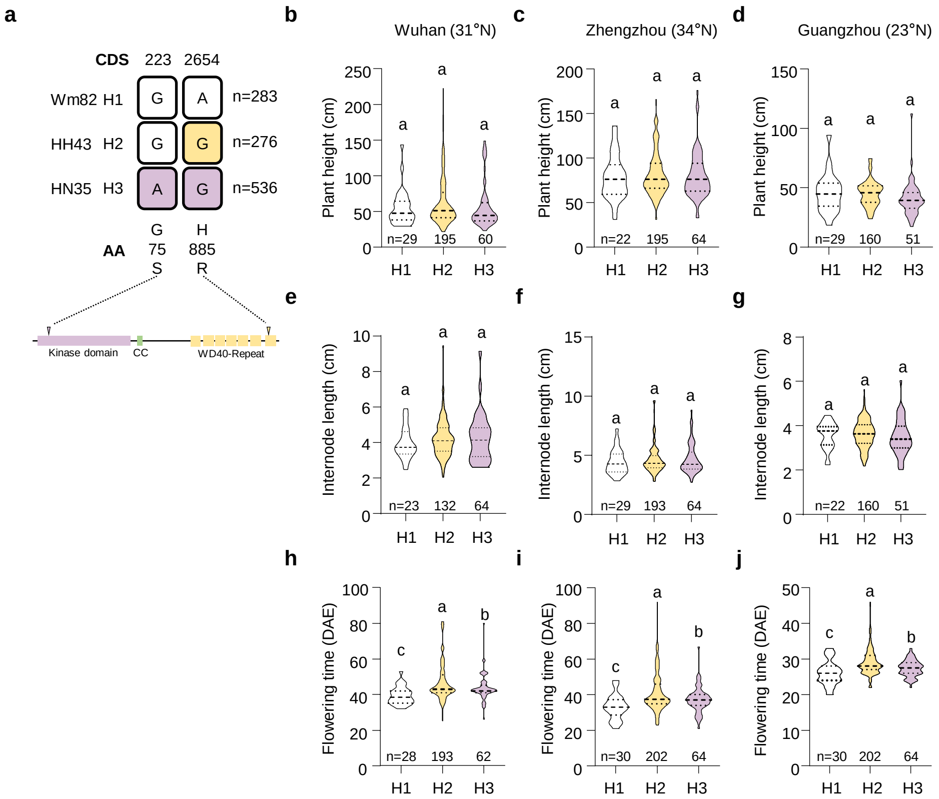

Supplementary Fig. 9 Haplotypes and phenotype correlation analysis of *RIN1*.

a Haplotypes of *RIN1*. CDS, coding region. AA, amino acid. n, the number of accessions with each haplotype. Haplotypes were extracted from the panel of 1,295 of sequenced accessions comprising 146 wild soybeans, 575 landraces and 574 improved cultivars as previously described[28]. The total number of accessions of three haplotypes are 1,095 due to the filtering of some poorly sequenced data. **b–d** Plant height (cm) of different haplotypes in *RIN1* in different regions in 2019, including Wuhan (**b**), Zhengzhou (**c**) and Guangzhou (**d**), China. **e–g** Internode length per plant (cm) of different haplotypes in *RIN1* in different regions in 2019, including Wuhan (**e**), Zhengzhou (**f**) and Guangzhou (**g**), China. **h–j** Flowering time (DAE) of different haplotypes in *RIN1* in different regions in 2019, including Wuhan (**h**), Zhengzhou (**i**) and Guangzhou (**j**), China. 297 accessions, including 137 landraces and 160 improved cultivars were used for correlation analysis in (**b**)–(**j**). The 297 accessions are listed in the Supplementary table 5 and phenotypes of 297 accessions are listed in the Source Data file. One-way ANOVA with Tukey's post-test was performed to determine statistically significant differences in (**b**)–(**j**), *P* <0.05. a, b and c letters indicate statistically significant differences.

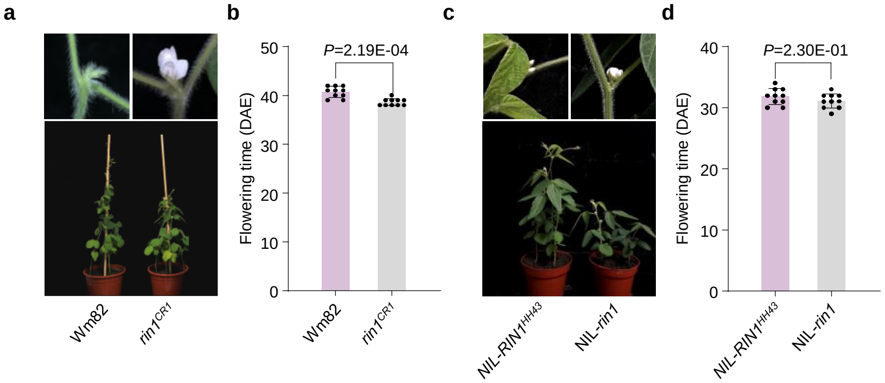

Supplementary Fig. 10 Flowering time of Wm82, *rin1*^{CR1} mutants and *RIN1*-NILs.
a Phenotypes of Wm82 and *rin1*^{CR1} at 38 DAE. **b** Flowering time (DAE) of Wm82 and *rin1*^{CR1}. **c** Phenotypes of NIL-*RIN1*^{HH43} and NIL-*rin1* at 30 DAE. **d** Flowering time (DAE) of NIL-*RIN1*^{HH43} and NIL-*rin1*. Plants in all panels were grown in growth chambers under long-day photoperiods (16 h light/8 h dark). All data are means ± SEM (n = 10 plants). A student's *t*-test (two-sided) was used to generate the *P* values in (**a**) and (**d**).

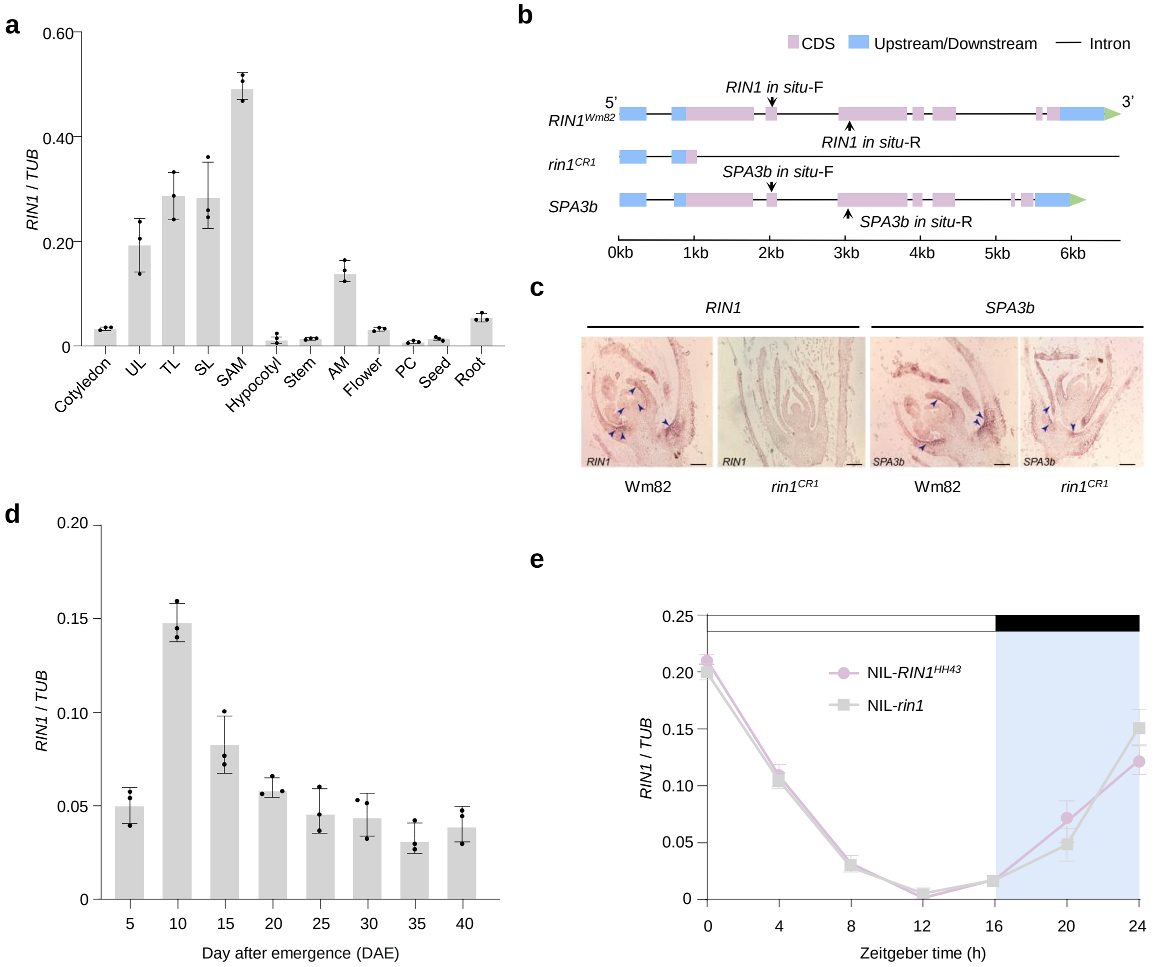

Supplementary Fig. 11 Expression pattern of *RIN1* in long-day photoperiods.

a Expression of *RIN1* in different tissues in Wm82. UL: Unifoliate leaf; TL: First trifoliolate leaf; SL: Second trifoliolate leaf; SAM: Shoot apical meristem; AM: Axillary meristem; PC: Pod coat. Data shown are relative to the control gene *Tubulin* (*TUB*). Data shown are means ± SD from three independent biological replicates. **b** Schematic representation of the positions of the *RIN1* and *SPA3b* probes used for *in situ* hybridization in (**c**). **c** *In situ* hybridization to determine the spatial expression pattern of *RIN1* and *SPA3b* in the stem tips of Wm82 and *rin1*^{CR1}. Arrows represent the expression positions of *RIN1* and *SPA3b*. Scale bars = 100 μm. **d** *RIN1* dynamics in the shoot apex at different stages in Wm82. Data shown are relative to the control gene *Tubulin* (*TUB*). Data shown are means ± SD from three independent biological replicates. **e** Relative expression levels of *RIN1* in NIL-*RIN1*^{HH43} and NIL-*rin1*. Data shown in are relative to the control gene *Tubulin* (*TUB*). Data shown are means ± SD from three independent biological replicates. All plants used in (**a**)–(**e**) were grown in growth chambers under long-day photoperiods (16 h light/8 h dark).

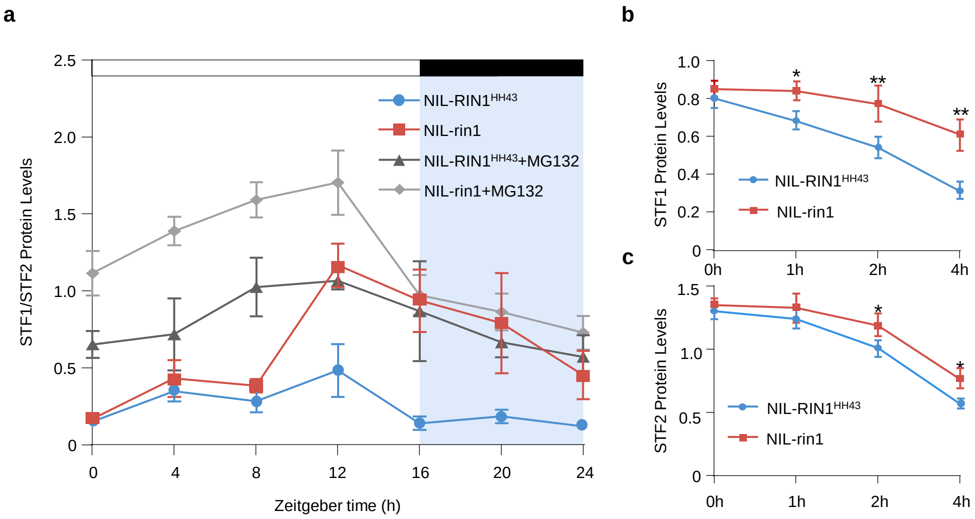

Supplementary Fig. 12 Quantitative analysis of relative STF protein abundance by ImageJ software.
a Quantitative analysis of relative STF1 and STF2 protein from **Fig. 3e**. **b,c** Quantitative analysis of relative STF1 (**b**) and STF2 protein (**c**) from **Fig. 3f**. Data shown in (**b**) and (**c**) are means ± SD from three independent biological replicates. A student's *t*-test (two-sided) was used to generate the *P* values of (**b**) and (**c**), $*P < 0.05$, $**P < 0.01$.

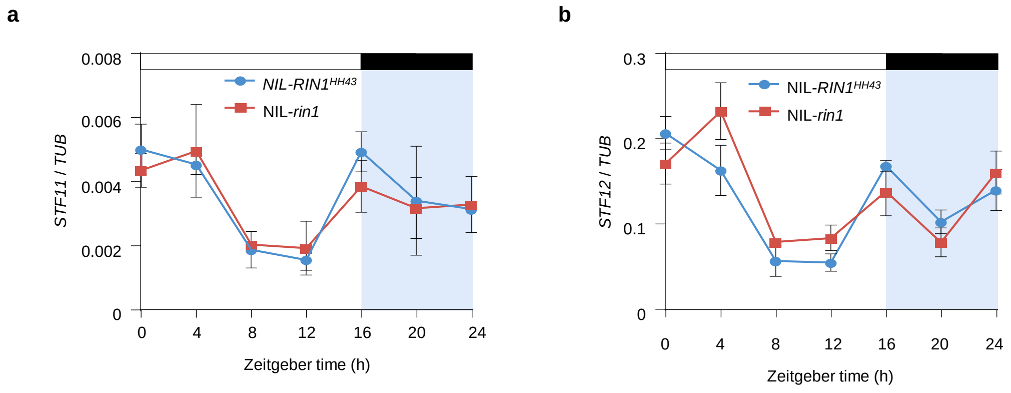

Supplementary Fig. 13 The *rin1* mutation does not lead to differential expression of *STF1* and *STF2*.
a–b Relative expression of *STF1* (**a**) and *STF2* (**b**) in the SAM of NILs-*RIN1* plants. Samples were harvested from ZT0 to ZT24 at 10 DAE. Data shown are relative to the control gene *Tubulin* (*TUB*). Data shown are means ± SD from three independent biological replicates. All plants in (**a**) and (**b**) were grown in growth chambers under long-day photoperiods (16 h light/8 h dark).

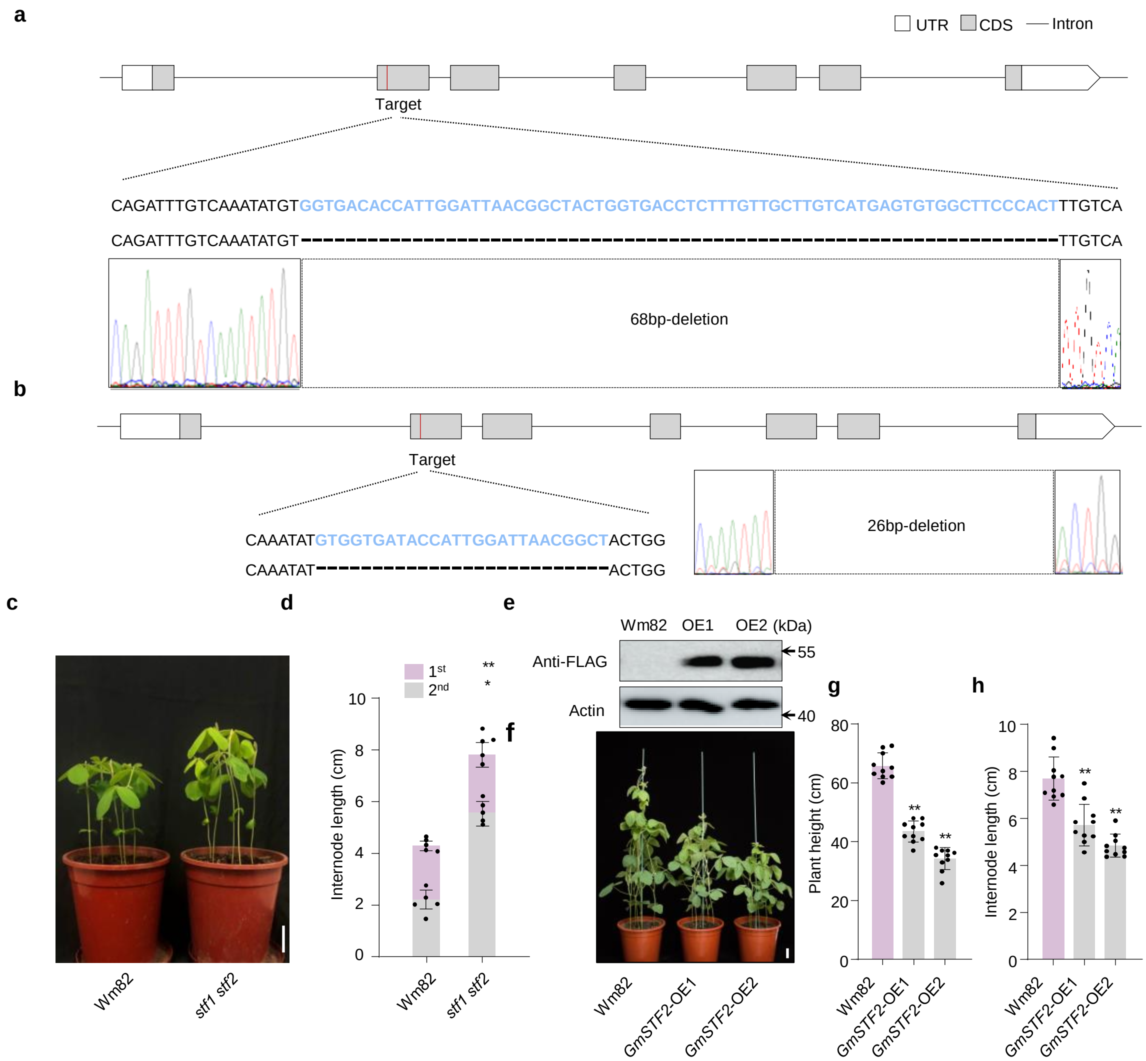

Supplementary Fig. 14 *STF1* and *STF2* genes regulate soybean internode length in long-day photoperiods.
a CRISPR/Cas9 induced a 68-bp deletion in *STF1* used to generate the *stf1 stf2* double mutant. The red lines indicate the CRISPR/Cas9 target that shares homology with *STF2*. **b** CRISPR/Cas9 induced a 26-bp deletion in *STF2* used to generate the *stf1 stf2* double mutant. The red lines indicate the CRISPR/Cas9 target that shares homology with *STF1*. The picture of (**a**) and (**b**) were generated by BioEdit. **c** Phenotypes of Wm82 and *stf1 stf2* double mutants under long-day photoperiods (16 h light/8 h dark). Scale bar = 5 cm. **d** Internode length of Wm82 and *stf1 stf2* double mutants under long-day photoperiods (16 h light/8 h dark). 1st, the first internode; 2nd, the second internode. Data are means ± SEM (n = 5 plants). A student's *t*-test (two-sided) was used to generate the *P* values, **P* <0.05, ***P* <0.01. **e** Western blot against the FLAG epitope in two genetically independent transgenic lines constitutively expressing p35S–STF2–3FLAG. Black arrows represent the positions of protein ladder. **f–h** Phenotypic differences between Wm82 and *STF2* overexpression lines. **f** Phenotypes of Wm82 and transgenic *STF2* overexpression lines at 30 DAE under long-day photoperiods (16 h light/8 h darkness). **g** Plant height (cm). **h** Internode length per plant (cm). All data in (**g**) and (**h**) are means ± SEM (n = 10 plants). A student's *t*-test (two-sided) was used to generate the *P* values, **P* <0.05, ***P* <0.01.

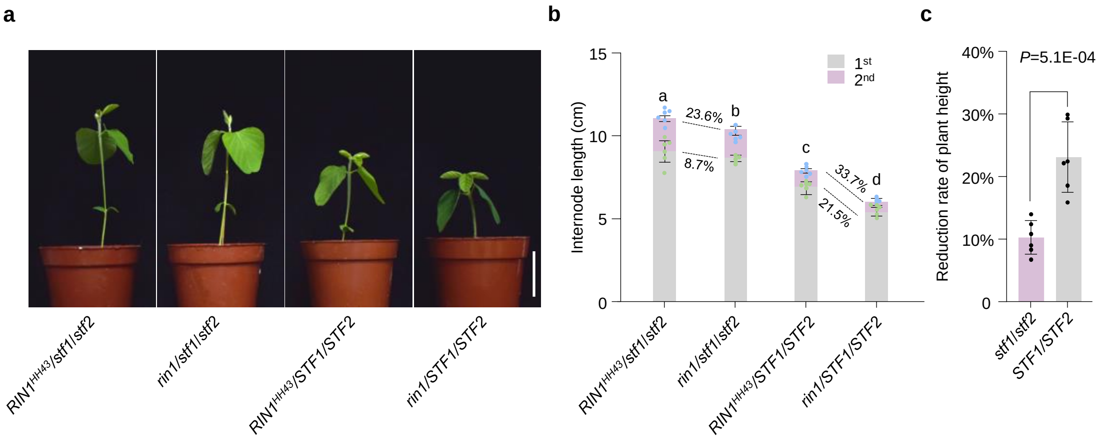

Supplementary Fig. 15 *RIN1* is partially dependent on *STF1* and *STF2* for internode-length regulation.
a Phenotypes of *RIN1^{HH43}/STF1/STF2*, *rin1/STF1/STF2*, *RIN1^{HH43}/stf1/stf2*, and *rin1/stf1/stf2* under long-day photoperiods (16 h light/8 h dark). Scale bar = 5 cm. **b** Internode length (cm) of *RIN1^{HH43}/STF1/STF2*, *rin1/STF1/STF2*, *RIN1^{HH43}/stf1/stf2*, and *rin1/stf1/stf2* under long-day photoperiods (16 h light/8 h dark). All data are means ± SEM (n = 6 plants). One-way ANOVA with Tukey's post-test was performed to determine statistically significant differences, $P < 0.05$. a, b, c and d letters indicate statistically significant differences. The green dots for 1st internode length and blue dots for 2nd internode length. **c** Significant differences analysis of the rate of plant height reduction in *rin1* under *STF1/STF2* and *stf1/stf2* backgrounds, respectively, in (**b**). All data are means ± SEM (n = 6 plants). A student's *t*-test (two-sided) was used to generate the *P* values.

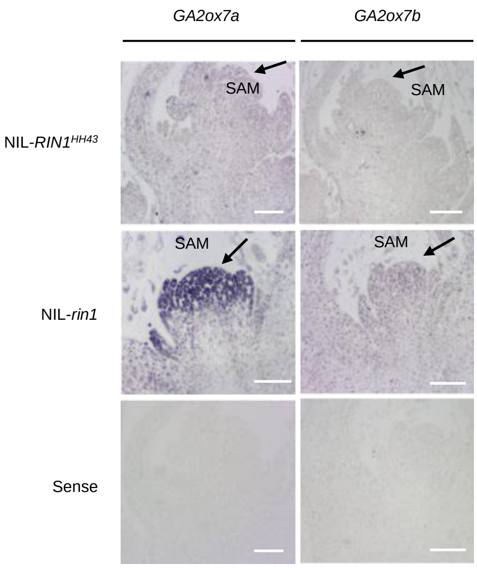

Supplementary Fig. 16 *In situ* hybridization of *GA2ox7a* and *GA2ox7b* in NIL-*RIN1*^{HH43} and NIL-*rin1.*
Spatial expression pattern of *GA2ox7a* and *GA2ox7b* in the stem tips of NIL-*RIN1*^{HH43} and NIL-*rin1* at 10 DAE under long-day photoperiods (16 h light/8 h dark) detected by *in situ* hybridization. Black arrows point to shoot apical meristems (SAMs). Scale bars = 100 μm. Three independent biological replicates were performed.

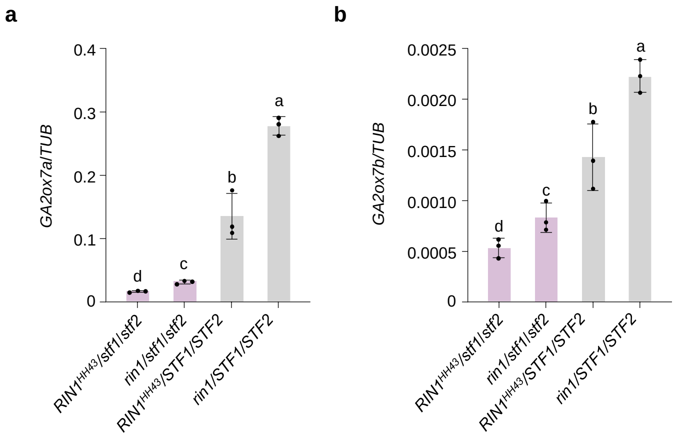

Supplementary Fig. 17 RT-qPCR further confirms that *RIN1* is partially dependent on *STF1* and *STF2* for internode-length regulation.

a,b Relative expression of *GA2ox7a* (**d**) and *GA2ox7b* (**e**) in the SAM of *RIN1^{HH43}/STF1/STF2*, *rin1/STF1/STF2*, *RIN1^{HH43}/stf1/stf2*, and *rin1/stf1/stf2* under long-day photoperiods (16 h light/8 h dark). Samples were harvested at ZT0 of 10 DAE. Data shown are relative to the control gene *Tubulin* (*TUB*). Data shown are means ± SD from three independent biological replicates. One-way ANOVA with Tukey's post-test was performed to determine statistically significant differences, P <0.05. a, b, c and d letters indicate statistically significant differences. Plants were grown in growth chambers under long-day photoperiods (16 h light/8 h dark).

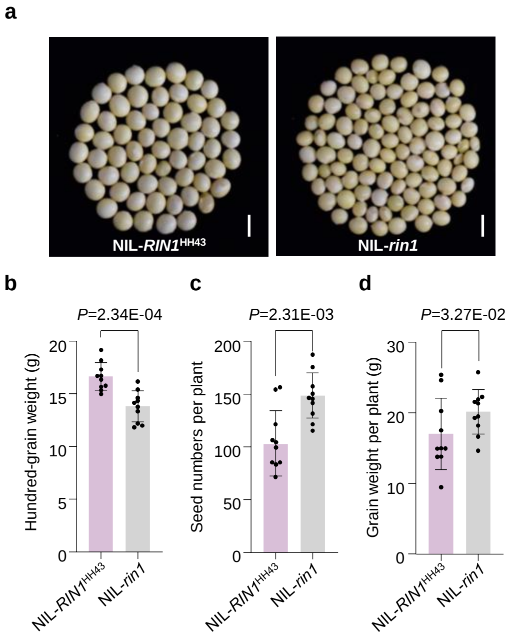

Supplementary Fig. 18 *RIN1* contributes to grain yield in field conditions.
a–d Yield traits of NIL-*RIN1*^{HH43} and NIL-*rin1* plants cultivated in a field in Shijiazhuang, China (37°27′N, 113°30′E) at a normal density under long-day natural photoperiods. **a** Representative images of whole seeds from NIL-*RIN1*^{HH43} (left) and NIL-*rin1* (right). Scale bar = 1 cm. **b** Hundred-grain weight (g); **c** Seed number per plant (g); **d** Grain weight per plant (g). All data of (**b**)–(**d**) are means ± SEM (n = 10 plants). A student's *t*-test (two-sided) was used to generate the *P* values.

Supplementary Table 1 QTLs for plant height detected in the HNH population.

QTL-LG	Year	Chr.	Detection method	Physical length (Wm82)	Max LOD	PVE%	ADD
RIN1	2017-F_2	12	Map-QTL	26,744,935-38,784,872	20.43	37.40	-13.53
RIN1	2018-F_3	12	Map-QTL	26,744,935-38,784,872	10.48	43.20	-12.68

PVE (%), percentage of phenotypic variance explained by the QTL.
ADD, The additive effects contributed by QTLs. Positive value (+) of the additive effect indicates the allele from *rin1* phenotype, negative value (-) of the additive effect indicates the allele originating from HH43 reduced phenotype.
Max LOD, maximum logarithm-of-odds (LOD) scores.

Supplementary Table 2 QTLs for internode length detected in the HNH population.

QTL-LG	Year	Chr.	Detection method	Physical length（Wm82）	Max LOD	PVE%	ADD
RIN1	2017-F_2	12	Map-QTL	26,744,935-38,784,872	13.83	27.20	-0.51
RIN1	2018-F_3	12	Map-QTL	26,744,935-38,784,872	11.10	43.30	-0.58

PVE (%), percentage of phenotypic variance explained by the QTL.
ADD, The additive effects contributed by QTLs. Positive value (+) of the additive effect indicates the allele from *rin1* phenotype, negative value (-) of the additive effect indicates the allele originating from HH43 reduced phenotype.
Max LOD, maximum logarithm-of-odds (LOD) scores.

Supplementary Table 3 Polymorphic variations of the predected genes between parents within CDS in the interval of RIN1 locus.

Gene ID	Gene Name	Position (Wm82 reference)	Changes of codon[a]	Changes of amino acid[b]	Mutation type	*rin1*-type	HH43-type	HN35-type
Glyma.12G224500	Protein kinase superfamily protein	38388858..38396091	-	-	-	-	-	-
Glyma.12G224600	SPA1-related 3	38408929..38415581	G223A	G75S	nonsynonymous SNV	1/1	0/0	1/1
			G2521T	E841X	stop gain	1/1	0/0	0/0
			A2654G	H885R	nonsynonymous SNV	1/1	1/1	1/1
Glyma.12G224700	Core-2/I-branching beta-1,6-N-acetylglucosaminyltransferase family protein	38418438..38422071	-	-	-	-	-	-
Glyma.12G224800	Proton pump interactor 1	38431962..38436035	G1381C	E461Q	nonsynonymous SNV	1/1	1/1	1/1
			T1643C	V548A	nonsynonymous SNV	1/1	1/1	1/1
Glyma.12G224900	RNA-binding S4 domain-containing protein	38438727..38445151	T8C	V3A	nonsynonymous SNV	1/1	1/1	1/1
Glyma.12G225000	Ribosomal protein L3 plastid	38447980..38452353	-	-	-	-	-	-
Glyma.12G225100	Stigma-specific Stig1 family protein	38454691..38456172	-	-	-	-	-	-

Changes of codon[a], number represents the SNP position in the CDS.
Changes of amino acid[b], number represents the amino acid position in protein.
type, 0/0 and 1/1 represented bases type same to reference (Glycine max Wm82.a2.v1) and mutation type, respectively.

Supplementary Table 4 Indel and single-nucleotide polymorphism (SNP) markers used to map-based cloning and NILs construction.

Primer ID	Position (Wm82)	Type	Primer Sequences (F/R)
ID122023	275665	Indel	TGCATGCAAAAATAAACACGA<br>AAAGGGTGGAGTGGGCAT
ID12028	317249	Indel	CAGCAAGGGGGAACAGAA<br>GTTTGTTGGCGGAGCAGT
ID12060	1123437	Indel	GGGTGGTTGGAGAAGCCT<br>TCATCTACGTTTTGGCGCT
ID12158	4971769	Indel	CCTGCATCCGCAAGATTC<br>CCGGAGACGGAGAAGGAT
ID12187	7210795	Indel	TCATCTTTTGAGGCCTTCTCA<br>CATGGGTTTGATGGAGTGC
ID12257	15571507	Indel	GAAACGGTGCCGTATTGG<br>ATCTCCCACACCACACGC
ID12332	20082725	Indel	GCGAAGCCGTTGAGAGAA<br>ACTCCCAAGCCTCCCATC
M1(ID12279)	27956411	Indel	CACTAATCAGGTGCTGCATC<br>CCCTTTATTCCTACTTCTACCAG
M2(ID12383)	38390241	Indel	TCCAAAATCTACTCTATGCG<br>GGTGTATTTGTTTGTTTGC
RIN1	38414709	SNP	CTTTGTGGGGTTATCAGTATCTGATGGTTACATTGCCACTGGTTTC<br>CTGCACAGCATCGTCCACTTCGTTGC
M3(ID12384)	38466548	Indel	GCACCCCAGGTTTATCATTG<br>CTATAGGTCTCTAGTGTGCCTC
M4 (ID12385)	38528916	Indel	ACAAACCCAGCATTTCGTG<br>GAGATGACATAATTATGTTTATCGTG
M5 (ID12386)	38666649	Indel	AGAATGTGGGCGTAGCTAG<br>GTTAGATCCCGGGAGTTC
M6 (ID12388)	38806992	Indel	TTATCTTTTGGGAAAATGAC<br>GTCCTATATATACTCTTGTC
M7 (ID12390)	39038815	Indel	CCTTTTGGATATATTCTTAC<br>TTGAAGTTAAGAAACCTGAG
M8 (ID12396)	39618541	Indel	AAAGCTCTCTCCCTTGGGGC<br>TTACCTCTAATTATTCCCTC

Supplementary Table 5 The 297 resequenced aceessions used for correlation analysis.

Haplotype	Accessions	Type
H1	GDC004	Improved cultivar
H1	GDC021	Landrace
H1	GDC034	Improved cultivar
H1	GDC037	Landrace
H1	GDC038	Improved cultivar
H1	GDC042	Improved cultivar
H1	GDC048	Improved cultivar
H1	GDC073	Improved cultivar
H1	GDC121	Improved cultivar
H1	GDC140	Improved cultivar
H1	GDC152	Improved cultivar
H1	GDC164	Improved cultivar
H1	GDC170	Improved cultivar
H1	GDC175	Improved cultivar
H1	GDC191	Improved cultivar
H1	GDC196	Improved cultivar
H1	GDC197	Improved cultivar
H1	GDC204	Improved cultivar
H1	GDC209	Improved cultivar
H1	GDC213	Improved cultivar
H1	GDC217	Improved cultivar
H1	GDC229	Improved cultivar
H1	GDL001	Landrace
H1	GDL104	Landrace
H1	GDL117	Landrace
H1	GDL127	Landrace
H1	GDL139	Landrace
H1	GDL140	Landrace
H1	GDL153	Landrace
H1	GDL180	Improved cultivar
H2	GDC003	Landrace
H2	GDC005	Landrace
H2	GDC008	Landrace
H2	GDC013	Improved cultivar
H2	GDC014	Improved cultivar
H2	GDC015	Landrace
H2	GDC017	Landrace
H2	GDC018	Landrace
H2	GDC019	Landrace
H2	GDC023	Landrace
H2	GDC024	Landrace
H2	GDC025	Landrace
H2	GDC026	Landrace
H2	GDC036	Landrace
H2	GDC039	Improved cultivar
H2	GDC040	Landrace
H2	GDC041	Landrace

Supplementary Table 5 The 297 resequenced aceessions used for correlation analysis.

Haplotype	Accessions	Type
H2	GDC044	Landrace
H2	GDC047	Landrace
H2	GDC050	Improved cultivar
H2	GDC051	Improved cultivar
H2	GDC053	Improved cultivar
H2	GDC054	Landrace
H2	GDC055	Landrace
H2	GDC058	Landrace
H2	GDC061	Landrace
H2	GDC066	Improved cultivar
H2	GDC067	Improved cultivar
H2	GDC068	Improved cultivar
H2	GDC071	Improved cultivar
H2	GDC074	Improved cultivar
H2	GDC075	Improved cultivar
H2	GDC078	Improved cultivar
H2	GDC079	Improved cultivar
H2	GDC081	Improved cultivar
H2	GDC082	Improved cultivar
H2	GDC083	Improved cultivar
H2	GDC084	Improved cultivar
H2	GDC085	Improved cultivar
H2	GDC086	Improved cultivar
H2	GDC088	Improved cultivar
H2	GDC090	Improved cultivar
H2	GDC091	Improved cultivar
H2	GDC092	Improved cultivar
H2	GDC094	Improved cultivar
H2	GDC096	Improved cultivar
H2	GDC097	Improved cultivar
H2	GDC098	Improved cultivar
H2	GDC101	Improved cultivar
H2	GDC102	Improved cultivar
H2	GDC104	Improved cultivar
H2	GDC105	Improved cultivar
H2	GDC106	Improved cultivar
H2	GDC107	Improved cultivar
H2	GDC108	Improved cultivar
H2	GDC110	Improved cultivar
H2	GDC111	Improved cultivar
H2	GDC113	Improved cultivar
H2	GDC114	Improved cultivar
H2	GDC118	Improved cultivar
H2	GDC119	Improved cultivar
H2	GDC120	Improved cultivar
H2	GDC122	Improved cultivar
H2	GDC123	Improved cultivar
H2	GDC124	Improved cultivar
H2	GDC125	Improved cultivar

Supplementary Table 5 The 297 resequenced aceessions used for correlation analysis.

Haplotype	Accessions	Type
H2	GDC127	Improved cultivar
H2	GDC130	Improved cultivar
H2	GDC131	Improved cultivar
H2	GDC132	Improved cultivar
H2	GDC134	Improved cultivar
H2	GDC137	Improved cultivar
H2	GDC138	Improved cultivar
H2	GDC139	Improved cultivar
H2	GDC141	Improved cultivar
H2	GDC142	Improved cultivar
H2	GDC145	Improved cultivar
H2	GDC146	Improved cultivar
H2	GDC147	Improved cultivar
H2	GDC150	Improved cultivar
H2	GDC153	Improved cultivar
H2	GDC154	Improved cultivar
H2	GDC155	Improved cultivar
H2	GDC156	Improved cultivar
H2	GDC158	Improved cultivar
H2	GDC160	Improved cultivar
H2	GDC161	Improved cultivar
H2	GDC162	Landrace
H2	GDC163	Improved cultivar
H2	GDC165	Improved cultivar
H2	GDC168	Improved cultivar
H2	GDC173	Improved cultivar
H2	GDC174	Improved cultivar
H2	GDC176	Improved cultivar
H2	GDC178	Improved cultivar
H2	GDC182	Improved cultivar
H2	GDC183	Improved cultivar
H2	GDC184	Improved cultivar
H2	GDC185	Improved cultivar
H2	GDC187	Improved cultivar
H2	GDC189	Improved cultivar
H2	GDC190	Improved cultivar
H2	GDC194	Improved cultivar
H2	GDC195	Improved cultivar
H2	GDC201	Improved cultivar
H2	GDC202	Improved cultivar
H2	GDC206	Improved cultivar
H2	GDC211	Improved cultivar
H2	GDC214	Improved cultivar
H2	GDC215	Improved cultivar
H2	GDC216	Improved cultivar
H2	GDC218	Improved cultivar
H2	GDC220	Landrace
H2	GDC221	Improved cultivar
H2	GDC226	Landrace

Supplementary Table 5 The 297 resequenced aceessions used for correlation analysis.

Haplotype	Accessions	Type
H2	GDC227	Landrace
H2	GDC228	Improved cultivar
H2	GDC230	Improved cultivar
H2	GDC231	Improved cultivar
H2	GDL002	Landrace
H2	GDL003	Landrace
H2	GDL006	Landrace
H2	GDL008	Landrace
H2	GDL014	Landrace
H2	GDL016	Landrace
H2	GDL019	Landrace
H2	GDL020	Landrace
H2	GDL024	Landrace
H2	GDL026	Landrace
H2	GDL029	Landrace
H2	GDL030	Landrace
H2	GDL034	Landrace
H2	GDL035	Landrace
H2	GDL040	Landrace
H2	GDL041	Landrace
H2	GDL042	Landrace
H2	GDL048	Landrace
H2	GDL049	Landrace
H2	GDL054	Landrace
H2	GDL055	Landrace
H2	GDL056	Landrace
H2	GDL057	Landrace
H2	GDL059	Landrace
H2	GDL063	Landrace
H2	GDL065	Landrace
H2	GDL067	Landrace
H2	GDL069	Landrace
H2	GDL070	Landrace
H2	GDL071	Landrace
H2	GDL073	Landrace
H2	GDL074	Landrace
H2	GDL076	Landrace
H2	GDL079	Landrace
H2	GDL080	Landrace
H2	GDL082	Landrace
H2	GDL083	Landrace
H2	GDL084	Landrace
H2	GDL088	Landrace
H2	GDL089	Landrace
H2	GDL091	Landrace
H2	GDL092	Improved cultivar
H2	GDL094	Landrace
H2	GDL099	Landrace
H2	GDL108	Landrace

Supplementary Table 5 The 297 resequenced aceessions used for correlation analysis.

Haplotype	Accessions	Type
H2	GDL110	Landrace
H2	GDL111	Landrace
H2	GDL120	Landrace
H2	GDL121	Landrace
H2	GDL125	Landrace
H2	GDL129	Landrace
H2	GDL133	Landrace
H2	GDL136	Landrace
H2	GDL138	Landrace
H2	GDL142	Landrace
H2	GDL143	Landrace
H2	GDL144	Landrace
H2	GDL145	Landrace
H2	GDL147	Landrace
H2	GDL151	Landrace
H2	GDL154	Landrace
H2	GDL156	Landrace
H2	GDL158	Landrace
H2	GDL159	Landrace
H2	GDL161	Landrace
H2	GDL165	Improved cultivar
H2	GDL166	Landrace
H2	GDL169	Landrace
H2	GDL170	Landrace
H2	GDL173	Landrace
H2	GDL175	Landrace
H2	GDL177	Landrace
H2	GDL181	Landrace
H2	GDL182	Landrace
H2	GDL183	Improved cultivar
H2	GDL184	Landrace
H2	GDL185	Landrace
H2	GDL186	Landrace
H2	GDL188	Landrace
H2	GDL190	Landrace
H2	GDL192	Landrace
H2	GDL197	Landrace
H2	GDL202	Landrace
H2	GDL204	Landrace
H3	GDC006	Improved cultivar
H3	GDC010	Landrace
H3	GDC012	Landrace
H3	GDC020	Improved cultivar
H3	GDC032	Landrace
H3	GDC043	Improved cultivar
H3	GDC046	Landrace
H3	GDC049	Improved cultivar
H3	GDC057	Improved cultivar
H3	GDC059	Improved cultivar

Supplementary Table 5 The 297 resequenced aceessions used for correlation analysis.

Haplotype	Accessions	Type
H3	GDC062	Improved cultivar
H3	GDC063	Improved cultivar
H3	GDC064	Improved cultivar
H3	GDC065	Improved cultivar
H3	GDC069	Improved cultivar
H3	GDC070	Improved cultivar
H3	GDC072	Improved cultivar
H3	GDC077	Improved cultivar
H3	GDC080	Improved cultivar
H3	GDC089	Improved cultivar
H3	GDC100	Improved cultivar
H3	GDC103	Improved cultivar
H3	GDC116	Improved cultivar
H3	GDC117	Improved cultivar
H3	GDC126	Improved cultivar
H3	GDC128	Improved cultivar
H3	GDC129	Improved cultivar
H3	GDC133	Improved cultivar
H3	GDC159	Improved cultivar
H3	GDC166	Improved cultivar
H3	GDC167	Improved cultivar
H3	GDC169	Improved cultivar
H3	GDC171	Improved cultivar
H3	GDC172	Improved cultivar
H3	GDC179	Improved cultivar
H3	GDC180	Improved cultivar
H3	GDC181	Improved cultivar
H3	GDC186	Improved cultivar
H3	GDC188	Improved cultivar
H3	GDC200	Improved cultivar
H3	GDC210	Improved cultivar
H3	GDC212	Improved cultivar
H3	GDC219	Improved cultivar
H3	GDC222	Landrace
H3	GDC225	Improved cultivar
H3	GDL004	Landrace
H3	GDL015	Landrace
H3	GDL033	Landrace
H3	GDL043	Landrace
H3	GDL058	Landrace
H3	GDL062	Landrace
H3	GDL064	Landrace
H3	GDL096	Landrace
H3	GDL102	Landrace
H3	GDL105	Improved cultivar
H3	GDL112	Landrace
H3	GDL124	Landrace
H3	GDL126	Landrace
H3	GDL131	Landrace
H3	GDL132	Landrace
H3	GDL155	Landrace
H3	GDL160	Landrace
H3	GDL176	Landrace
H3	GDL195	Landrace

Supplementary Table 6 Primers used in the study.

**Gene cloning 5'-3'**	
RIN1-Outside-F	CTGTTGTGAGGTTGGCCTAGC
RIN1-Outside-R	GGTTCTGTCACCTCATGCTC
RIN1-F	ATGTGTTGTTTTACTTGGCC
RIN1-R	TTAAACCATCTCCAGAATTTTGAC
STF1-F	ATGGAACGAAGTGGCGGAATG
STF1-R	CTCAGCATTATTGGTACCAT
STF2-F	ATGGAACGAAGTGGCGGAATG
STF2-R	CTCAGCATTATTGGTACCAC
**CRISPR/Cas9 construction 5'-3'**	
RIN1-At3d-T1F1	gtcaTCTAGGGCCTTGAATAGTTC
RIN1-At3d-T1R1	aaacGAACTATTCAAGGCCCTAGA
RIN1-At3b-T2F2	gtcaAAGGGTTCATTGTCCTCAA
RIN1-At3b-T2R2	aaacTTGAGGACAATGAACCCTT
RIN1-At6-1-T3F3	attgCAGCGATAGAGCTGAGACAA
RIN1-At6-1-T3R3	aaacTTGTCTCAGCTCTATCGCTG
**Mutants sequencing primers 5'-3'**	
RIN1-cas9test-F1	CTGAAGAGTTTGTGAATCCATGG
RIN1-cas9test-R1	GATGACATGACGAAGCAGGAAGG
RIN1-cas9test-F2	CAAGTGCCTGACTGATCAAACC
RIN1-cas9test-R2	AGCTGACTCAAGTTTCTTAA
STF1-cas9test-F	TGAGTGATTTTCTGATCGTGATGC
STF1-cas9test-R	TCCGATGAGGGAAAGACCAA
STF2-cas9test-F	AAGAGATTGAACTGCATAGGCT
STF2-cas9test-R	CTTGCACTGGGGACAAGACT
**qRT-PCR 5'-3'**	
RIN1-RT-F	GGAGTTGTTGCAGAGCGAG
RIN1-RT-R	GAAACTTGATGCTGAACGCT
STF1-RT-F	ACACGGTTCTGACAGTGGGTCTAAA
STF1-RT-R	AAGCCACACTCATGACAAGCAACAA
STF2-RT-F	ACATAATGTCAAAAGATGGCAGAG
STF2-RT-R	CATTGTATTCCAAACGCACACCTA
GA2ox7a-qF	TGTAAAACCAAACCCTGAT
GA2ox7a-qR	AACTCTATGCTCCACACTCTT
GA2ox7b-qF	ACGGTCTCATGCCACATACT
GA2ox7b-qR	TTAACTGCGATCCATTTGCT
TUB-F	CTACACCGTTGGCAAAGAGA
TUB-R	GAAGACGAGGAAGCCTTGTAG
**In situ hybridization probe 5'-3'**	
RIN1-positive-F	GATTTAGGTGACACTATAGAATGCTCTGGGGAGTTGTTGCAGAGCGAG
RIN1-positive-R	TGTAATACGACTCACTATAGGGGAAACTTGATGCTGAACGCT
SPA3b-positive-F	GATTTAGGTGACACTATAGAATGCTCGGCAGGGAGTTGTTGCAGAGCGAC
SPA3b-positive-R	TGTAATACGACTCACTATAGGGGGAAACTTGATGCTGAATG
GA2ox7a-positive-F	GATTTAGGTGACACTATAGAATGCTTGTAAAACCAAACCCTGAT
GA2ox7a-positive-R	TGTAATACGACTCACTATAGGGAACTCTATGCTCCACACTCTT
GA2ox7b-positive-F	GATTTAGGTGACACTATAGAATGCTACGGTCTCATGCCACATACT
GA2ox7b-positive-R	TGTAATACGACTCACTATAGGGTTAACTGCGATCCATTTGCT

Supplementary Table 6 Primers used in the study.

**Y2H vector construction 5'-3'**	
RIN1-AD-ECOR1-F	GCCATGGAGGCCAGTGAATTCATGTGTTGTTTTACTTGGCCTAC
RIN1-AD-BamH1-R	CAGCTCGAGCTCGATGGATCCTTAAACCATCTCCAGAATTTTGAC
STF1-BD-ECOR1-F	ATGGCCATGGAGGCCGAATTCATGGAACGAAGTGGCGGAATG
STF1-BD-BamH1-R	CAGCTCGAGCTCGATGGATCCTTACTCAGCATTATTGGTACCAT
STF2-BD-ECOR1-F	ATGGCCATGGAGGCCGAATTCATGGAACGAAGTGGCGGAATG
STF2-BD-BamH1-R	CAGCTCGAGCTCGATGGATCCTTACTCAGCATTATTGGTACCAT
**Pull down assay and Cell free assay 5'-3'**	
RIN1-GST-F	CGCGGATCCATGTGTTGTTTTACTTGGCCTAC
RIN1-GST-R	AACCATCTCCAGAATTTTGACGAATTCCGG
STF1-MBP-F	TCCATGGGCGGCCGCGATATCATGGAACGAAGTGGCGGAATG
STF1-MBP-R	ACCTGCAGGGAATTCGGATCCTTACTCAGCATTATTGGTACCAT
STF2-MBP-F	TCCATGGGCGGCCGCGATATCATGGAACGAAGTGGCGGAATG
STF2-MBP-R	ACCTGCAGGGAATTCGGATCCTTACTCAGCATTATTGGTACCAC
**BIFC assay 5'-3'**	
RIN1-YCE-F	CCTGGCGCGCCACTAGTGGATCCATGTGTTGTTTTACTTGGCCTAC
RIN1-YCE-R	CATCCCGGGAGCGGTACCAACCATCTCCAGAATTTTGAC
STF1-YNE-F	ACGGGGGACTCTAGAATGGAACGAAGTGGCGGAATG
STF1-YNE-R	AGCGGTACCCTCGAGCTCAGCATTATTGGTACCAT
STF2-YNE-F	ACGGGGGACTCTAGAATGGAACGAAGTGGCGGAATG
STF2-YNE-R	AGCGGTACCCTCGAGCTCAGCATTATTGGTACCAC
PRR5a-YCE-F	CCTGGCGCGCCACTAGTGGATCCATGCCAGAGGTGGTGAT
PRR5a-YCE-R	CATCCCGGGAGCGGTACCTGCTCTCCTCTCCAG
PRR5a-YNE-F	ACGGGGGACTCTAGAATGCCAGAGGTGGTGAT
PRR5s-YNE-R	AGCGGTACCCTCGAGTGCTCTCCTCTCCAG
**Transient expression assay**	
pGA2ox7a-F	GTCGACGGTATCGATAAGCTTGGTAGCCGACATGGATCGA
pGA2ox7a-R	CGCTCTAGAACTAGTGGATCCAGAAGGAGACTAGCCACCGAGT
pGA2ox7b-F	GTCGACGGTATCGATAAGCTTCAAATATTGCTAGCCAGTAGCCAGC
pGA2ox7b-R	CGCTCTAGAACTAGTGGATCCTCTATCTTCTGTGATAGAAATTAGAAAAAAGC
