# Supplementary material for: Soybean reduced internode 1 determines internode length and improves grain yield at dense planting
Source: Nat Commun. 2023 Dec 1;14:7939. doi: 10.1038/s41467-023-42991-z (PMC10692089; doi:10.1038/s41467-023-42991-z)
Supplement: Supplementary file 3 — Reporting Summary [file 41467_2023_42991_MOESM3_ESM.pdf]

nature portfolio

Corresponding author(s): *Double-anonymous peer review submissions: write DAPR and your manuscript number here instead of author names.*

Last updated by author(s): *YYYY-MM-DD*

Reporting Summary

Nature Portfolio wishes to improve the reproducibility of the work that we publish. This form provides structure for consistency and transparency in reporting. For further information on Nature Portfolio policies, see our Editorial Policies and the Editorial Policy Checklist.

Statistics

For all statistical analyses, confirm that the following items are present in the figure legend, table legend, main text, or Methods section.

n/a	Confirmed	
☐	☒	The exact sample size (*n*) for each experimental group/condition, given as a discrete number and unit of measurement
☐	☒	A statement on whether measurements were taken from distinct samples or whether the same sample was measured repeatedly
☐	☒	The statistical test(s) used AND whether they are one- or two-sided<br>*Only common tests should be described solely by name; describe more complex techniques in the Methods section.*
☒	☐	A description of all covariates tested
☒	☐	A description of any assumptions or corrections, such as tests of normality and adjustment for multiple comparisons
☐	☒	A full description of the statistical parameters including central tendency (e.g. means) or other basic estimates (e.g. regression coefficient) AND variation (e.g. standard deviation) or associated estimates of uncertainty (e.g. confidence intervals)
☐	☒	For null hypothesis testing, the test statistic (e.g. *F*, *t*, *r*) with confidence intervals, effect sizes, degrees of freedom and *P* value noted<br>*Give P values as exact values whenever suitable.*
☒	☐	For Bayesian analysis, information on the choice of priors and Markov chain Monte Carlo settings
☒	☐	For hierarchical and complex designs, identification of the appropriate level for tests and full reporting of outcomes
☒	☐	Estimates of effect sizes (e.g. Cohen's *d*, Pearson's *r*), indicating how they were calculated

Our web collection on statistics for biologists contains articles on many of the points above.

Software and code

Policy information about availability of computer code

Data collection	The Nucleic acid sequences were aligned by DNAMAN 6 and the amino acid sequences were aligned by ClustalW Multiple alignments in DNAMAN 6;<br>The BIFC of RIN1 and STF1 and STF2 were acquried by Zeiss LSM 800 Confocal Laser Scanning Microcopy;<br>The western blot analysis were acquired by Amersham Imager 680.<br>The phenotypes of the plants were acquired by the Canon EOS 90D APS-C.
Data analysis	The bar charts and line charts were drawed by Graphpad Prism V8.0.1;<br>The qRT-PCR data was acquired by LightCycler®480 Ⅱ ;<br>The whole-genome SNP calling was performed by Genome Analysis Toolkit GATKv3.7-0;<br>Quantitation of protein abundance were analyzed using ImageJ V2.3.0;<br>The significance analysis results were acquired by SPSS 22.0.

For manuscripts utilizing custom algorithms or software that are central to the research but not yet described in published literature, software must be made available to editors and reviewers. We strongly encourage code deposition in a community repository (e.g. GitHub). See the Nature Portfolio guidelines for submitting code & software for further information.

Data

Policy information about availability of data

All manuscripts must include a data availability statement. This statement should provide the following information, where applicable:

- Accession codes, unique identifiers, or web links for publicly available datasets
- A description of any restrictions on data availability
- For clinical datasets or third party data, please ensure that the statement adheres to our policy

All data used in this work are available within the paper and supplementary files.;
The sequencing data used for phenotype association analysis were from Lu et al., 2020 while the phenotype data used for association analysis were listed in the Source data files;
All soybean nucleic acid and Amino acid sequences were obtained from Phytozome V13 (https://phytozome-next.jgi.doe.gov/);
All Amino acid sequences listed in the Supplementary Table 6 are obtained from phytozome V13 (https://phytozome-next.jgi.doe.gov/) and LIS: Legume Information System (https://www.legumeinfo.org/).

Research involving human participants, their data, or biological material

Policy information about studies with human participants or human data. See also policy information about sex, gender (identity/presentation), and sexual orientation and race, ethnicity and racism.

Reporting on sex and gender	N/A
Reporting on race, ethnicity, or other socially relevant groupings	N/A
Population characteristics	N/A
Recruitment	N/A
Ethics oversight	N/A

Note that full information on the approval of the study protocol must also be provided in the manuscript.

Field-specific reporting

Please select the one below that is the best fit for your research. If you are not sure, read the appropriate sections before making your selection.

[x] Life sciences [ ] Behavioural & social sciences [ ] Ecological, evolutionary & environmental sciences

For a reference copy of the document with all sections, see nature.com/documents/nr-reporting-summary-flat.pdf

Life sciences study design

All studies must disclose on these points even when the disclosure is negative.

Sample size	The sample size was determined based on preliminary data demonstrating statistically significant differences for each assay.
Data exclusions	No data were excluded from the analysis.
Replication	The replication information of each experiment was described in the figure legends.
Randomization	The samples in all the expression and phenotyping detection were randomly sampled.
Blinding	Blinding was not relevant as all processing methods were done through available software with consistent parameters utilized across all soybean plants and required controls.

Reporting for specific materials, systems and methods

We require information from authors about some types of materials, experimental systems and methods used in many studies. Here, indicate whether each material, system or method listed is relevant to your study. If you are not sure if a list item applies to your research, read the appropriate section before selecting a response.

Materials & experimental systems

n/a	Involved in the study	
☐	☒	Antibodies
☒	☐	Eukaryotic cell lines
☒	☐	Palaeontology and archaeology
☒	☐	Animals and other organisms
☒	☐	Clinical data
☒	☐	Dual use research of concern
☐	☒	Plants

Methods

n/a	Involved in the study	
☒	☐	ChIP-seq
☒	☐	Flow cytometry
☒	☐	MRI-based neuroimaging

Antibodies

Antibodies used

Commercial antibodies:anti-Flag (Abmart, M20008L, 1:2500 dilution), anti-Actin (Sigma, 2522, 1:1000 dilution), anti-MBP(Sigma, SAB2108749, 1:4000 dilution).

Validation

anti-FLAG (http://www.ab-mart.com.cn/ upload/20211217182008xz.pdf);
anti-MBP (https://www.sigmaaldrich.cn/CN/zh/product/sigma/sab2108749);
anti-Actin (https://www.sigmaaldrich.cn/CN/zh/product/sigma/a2522).

Dual use research of concern

Policy information about dual use research of concern

Hazards

Could the accidental, deliberate or reckless misuse of agents or technologies generated in the work, or the application of information presented in the manuscript, pose a threat to:

No	Yes	
☒	☐	Public health
☒	☐	National security
☒	☐	Crops and/or livestock
☒	☐	Ecosystems
☒	☐	Any other significant area

Experiments of concern

Does the work involve any of these experiments of concern:

No	Yes	
☒	☐	Demonstrate how to render a vaccine ineffective
☒	☐	Confer resistance to therapeutically useful antibiotics or antiviral agents
☒	☐	Enhance the virulence of a pathogen or render a nonpathogen virulent
☒	☐	Increase transmissibility of a pathogen
☒	☐	Alter the host range of a pathogen
☒	☐	Enable evasion of diagnostic/detection modalities
☒	☐	Enable the weaponization of a biological agent or toxin
☒	☐	Any other potentially harmful combination of experiments and agents
