# Supplementary material for: Soybean reduced internode 1 determines internode length and improves grain yield at dense planting
Source: Nat Commun. 2023 Dec 1;14:7939. doi: 10.1038/s41467-023-42991-z (PMC10692089; doi:10.1038/s41467-023-42991-z)
Supplement: Supplementary file 4 — Description of Additional Supplementary Files [file 41467_2023_42991_MOESM4_ESM.pdf]

Description of Additional Supplementary Files:

Supplementary Data 1: The sequencing data of RIN1 homologous gene SPA1a at RIN1 CRISPR/CAS9 knockout targets, Target1 and Target2, in rin1CR mutants.

Supplementary Data 2: The sequencing data of RIN1 homologous gene SPA1a at RIN1 CRISPR/CAS9 knockout targets, Target1 and Target2, in Wm82.

Supplementary Data 3: The sequencing data of RIN1 homologous gene SPA1b at RIN1 CRISPR/CAS9 knockout targets, Target1 and Target2, in rin1CR mutants.

Supplementary Data 4: The sequencing data of RIN1 homologous gene SPA1b at RIN1 CRISPR/CAS9 knockout targets, Target1 and Target2, in Wm82.

Supplementary Data 5: The sequencing data of RIN1 homologous gene SPA2a at RIN1 CRISPR/CAS9 knockout targets, Target1 and Target2, in rin1CR mutants.

Supplementary Data 6: The sequencing data of RIN1 homologous gene SPA2a at RIN1 CRISPR/CAS9 knockout targets, Target1 and Target2, in Wm82.

Supplementary Data 7: The sequencing data of RIN1 homologous gene SPA2c at RIN1 CRISPR/CAS9 knockout targets, Target1 and Target2, in rin1CR mutants.

Supplementary Data 8: The sequencing data of RIN1 homologous gene SPA2c at RIN1 CRISPR/CAS9 knockout targets, Target1 and Target2, in Wm82.

Supplementary Data 9: The sequencing data of RIN1 homologous gene SPA2d at RIN1 CRISPR/CAS9 knockout targets, Target1 and Target2, in rin1CR mutants.

Supplementary Data 10: The sequencing data of RIN1 homologous gene SPA2d at RIN1 CRISPR/CAS9 knockout targets, Target1 and Target2, in Wm82.

Supplementary Data 11: The sequencing data of RIN1 homologous gene SPA3b at RIN1 CRISPR/CAS9 knockout targets, Target1 and Target2, in rin1CR mutants.

Supplementary Data 12: The sequencing data of RIN1 homologous gene SPA3b at RIN1 CRISPR/CAS9 knockout targets, Target1 and Target2, in Wm82.

Supplementary Data 13: The sequencing data of RIN1 homologous gene SPA1a at RIN1 CRISPR/CAS9 knockout target, Target3, in rin1CR mutants.

Supplementary Data 14: The sequencing data of RIN1 homologous gene SPA1a at RIN1 CRISPR/CAS9 knockout target, Target3, in Wm82.

Supplementary Data 15: The sequencing data of RIN1 homologous gene SPA1b at RIN1 CRISPR/CAS9 knockout target, Target3, in rin1CR mutants.

Supplementary Data 16: The sequencing data of RIN1 homologous gene SPA1b at RIN1 CRISPR/CAS9 knockout target, Target3, in Wm82.

Supplementary Data 17: The sequencing data of RIN1 homologous gene SPA2a at RIN1 CRISPR/CAS9 knockout target, Target3, in rin1CR mutants.

Supplementary Data 18: The sequencing data of RIN1 homologous gene SPA2a at RIN1 CRISPR/CAS9 knockout target, Target3, in Wm82.

Supplementary Data 19: The sequencing data of RIN1 homologous gene SPA2c at RIN1 CRISPR/CAS9 knockout target, Target3, in rin1CR mutants.

Supplementary Data 20: The sequencing data of RIN1 homologous gene SPA2c at RIN1 CRISPR/CAS9 knockout target, Target3, in Wm82.

Supplementary Data 21: The sequencing data of RIN1 homologous gene SPA2dat RIN1 CRISPR/CAS9 knockout target, Target3, in rin1CR mutants.

Supplementary Data 22: The sequencing data of RIN1 homologous gene SPA2d at RIN1 CRISPR/CAS9 knockout target, Target3, in Wm82.

Supplementary Data 23: The sequencing data of RIN1 homologous gene SPA3b at RIN1 CRISPR/CAS9 knockout target, Target3, in rin1CR mutants.

Supplementary Data 24: The sequencing data of RIN1 homologous gene SPA3b at RIN1 CRISPR/CAS9 knockout target, Target3, in Wm82.

Supplementary Data 25: The sequencing data of RIN1 homologous gene SPA3c at RIN1 CRISPR/CAS9 knockout target, Target3, in rin1CR mutants.

Supplementary Data 26: The sequencing data of RIN1 homologous gene SPA3c at RIN1 CRISPR/CAS9 knockout target, Target3, in Wm82.

Supplementary Data 27: The sequencing data of RIN1 homologous gene SPA3d at RIN1 CRISPR/CAS9 knockout target, Target3, in rin1CR mutants.

Supplementary Data 28: The sequencing data of RIN1 homologous gene SPA3d at RIN1 CRISPR/CAS9 knockout target, Target3, in Wm82.
